# Hybrid Nanopore-Illumina Assemblies for Five Extraintestinal Pathogenic Escherichia coli Isolates

**DOI:** 10.1128/MRA.01027-20

**Published:** 2021-02-11

**Authors:** D. Mattrasingh, A. Hinz, L. Phillips, A. C. Carroll, A. Wong

**Affiliations:** a Department of Biology, Carleton University, Ottawa, Canada; Broad Institute

## Abstract

Extraintestinal pathogenic Escherichia coli (ExPEC) is an important source of multidrug-resistant infections, particularly in hospitals. We report hybrid Nanopore-Illumina assemblies for 5 ExPEC isolates with various drug resistance profiles.

## ANNOUNCEMENT

Extraintestinal pathogenic Escherichia coli (ExPEC) causes serious illnesses, including blood and urinary tract infections (UTIs), and has a wide variety of antibiotic resistance profiles (1). Five ExPEC isolates with various degrees of documented antibiotic resistance were collected as part of the CANWARD survey of antibiotic-resistant pathogens in Canada (2, 3) (kindly shared by the Zhanel laboratory). Two isolates were derived from UTIs (PB3 and PB4), two from blood infections (PB29 and PB35), and one from a respiratory infection (PB33). Isolates were collected under University of Manitoba Research Ethics Board approval (H2009:O59).

Strains were cultured overnight at 37°C in lysogeny broth (1% tryptone, 0.5% yeast extract, and 1% NaCl), and DNA was extracted using the One-4-All genomic DNA miniprep kit (BioBasic, Markham, Canada). Short reads were generated on the Illumina NextSeq platform using 150-bp paired-end reads with Nextera XT library preparation, generating a total of 162,925,724 clusters passing filter. Long reads were generated on the Nanopore MinION platform using the rapid barcoding kit and Guppy v3.1.5 base calling, generating a total of 697,371 reads and a mean read length of 1,176 bases. Quality scoring before and after trimming was performed using FastQC v0.11.7 (https://www.bioinformatics.babraham.ac.uk/projects/fastqc/), and Illumina reads were trimmed using Trimmomatic v0.38 (4) with the parameters LEADING:3 TRAILING:3 SLIDINGWINDOW:4:15 MINLEN:36. Porechop v.0.2.4 (https://github.com/rrwick/Porechop) was used to remove adapter sequences from the Nanopore reads. Hybrid assemblies were generated using the SPAdes optimizer Unicycler v0.4.8 (5). QUAST v4.6 (6) was used to produce summary statistics for the assembly. Genome annotations were carried out with the NCBI Prokaryotic Genome Annotation Pipeline v4.12 (7), serotypes were predicted using SeroTypeFinder v2.0.1 (8), and antimicrobial resistance (AMR) profiles were predicted with ResFinder v3.1 (9). Default parameter settings were used for all software unless otherwise indicated.

The genomes varied between 4,880,873 bp (PB4) and 5,309,474 bp (PB29) in length and maintained a GC content from 50.6 to 50.85 (Table 1). The strains harbored similar numbers of coding DNA sequences (CDS), rRNAs, and tRNAs. PB4 and PB29 carried two predicted CRISPR sequences each, while the others carried none. The strains carried between 0 and 6 chromosomal resistance mutations and between 1 and 14 resistance genes (Table 1). PB33 and PB35 both belong to serotype O25:H4-ST131, a major epidemic clone of ExPEC (10, 11).

**TABLE 1:** Assembly and genome characteristics for 5 ExPEC isolates

Strain	Total length (bp)	No. of contigs	Largest contig (bp)	Contig *N*_50_	Coverage^*a*^	No. of protein CDS^*b*^	No. of ARG^*c*^	No. of ARM^*d*^	ESBL^*e*^	Serotype
PB3	5,224,675	48	4,043,831	4,043,831	104; 23	4,793	2	0	None	O6:H1
PB4	4,880,873	23	1,168,174	688,147	70; 31	4,414	1	1	None	O−:H9
PB29	5,309,474	55	1,155,588	473,125	57; 15	4,909	13	0	CTX-M-15	O−:H9
PB33	5,168,956	27	1,099,130	710,413	25; 45	4,772	8	6	CTX-M-15	O25:H4
PB35	5,236,721	41	871,437	497,725	34; 28	4,792	14	5	CTX-M-15	O25:H4

a Mean fold coverage for Illumina reads; Nanopore reads, rounded to the nearest whole number.

b Predicted protein coding sequences, excluding pseudogenes.

c Number of predicted antibiotic resistance genes (ARG).

d Number of predicted antibiotic resistance mutations (ARM).

e Type (if any) of extended-spectrum beta-lactamase (ESBL) gene present in the genome.

We have provided *de novo* hybrid genome assemblies of five extraintestinal pathogenic E. coli isolates. Three of the isolates exhibit a genomic signature of multidrug resistance characterized by 14 or more resistance mutations/genes (PB29, PB33, and PB35). The remaining two isolates have relatively fewer resistance determinants (PB3 and PB4). These genomes will support future investigations into E. coli pathogenesis and resistance evolution.

### Data availability.

The assemblies and raw sequence data have been deposited in GenBank under BioProject PRJNA648312, with accession numbers JACFYA000000000 (assembly number GCA_014042275.1), JACFYB000000000 (GCA_014042265.1), JACFYC000000000 (GCA_014042305.1), JACFYD000000000 (GCA_014042325.1), and JACFYE000000000 (GCA_014265895.1). The versions described in this paper are the first versions, JACFYA010000000 to JACFYE010000000.
